# eHealth Literacy Among College Students: A Systematic Review With Implications for eHealth Education

**DOI:** 10.2196/jmir.1703

**Published:** 2011-12-01

**Authors:** Michael Stellefson, Bruce Hanik, Beth Chaney, Don Chaney, Bethany Tennant, Enmanuel Antonio Chavarria

**Affiliations:** ^1^Department of Health Education and BehaviorUniversity of FloridaGainesville, FLUnited States; ^2^Department of Health and KinesiologyTexas A&M UniversityCollege Station, TXUnited States

**Keywords:** eHealth literacy, college students, health occupations, professional preparation

## Abstract

**Background:**

eHealth literacy refers to the ability of individuals to seek, find, understand, and appraise health information from electronic resources and apply such knowledge to addressing or solving a health problem. While the current generation of college students has access to a multitude of health information on the Internet, access alone does not ensure that students are skilled at conducting Internet searches for health information. Ensuring that college students have the knowledge and skills necessary to conduct advanced eHealth searches is an important responsibility particularly for the medical education community. It is unclear if college students, especially those in the medical and health professions, need customized eHealth literacy training for finding, interpreting, and evaluating health- and medical-related information available on the Internet.

**Objective:**

The objective of our review was to summarize and critically evaluate the evidence from existing research on eHealth literacy levels among college students between the ages of 17 and 26 years attending various 4-year colleges and universities located around the world.

**Methods:**

We conducted a systematic literature review on numerous scholarly databases using various combinations of relevant search terms and Boolean operators. The records were screened and assessed for inclusion in the review based on preestablished criteria. Findings from each study that met inclusion criteria were synthesized and summarized into emergent themes.

**Results:**

In the final review we analyzed 6 peer-reviewed articles and 1 doctoral dissertation that satisfied the inclusion criteria. The number of participants in each reviewed study varied widely (from 34 to 5030). The representativeness of the results from smaller studies is questionable. All studies measured knowledge and/or behaviors related to college student ability to locate, use, and evaluate eHealth information. These studies indicated that many college students lack eHealth literacy skills, suggesting that there is significant room for improvement in college students’ ability to obtain and evaluate eHealth information.

**Conclusion:**

Although college students are highly connected to, and feel comfortable with, using the Internet to find health information, their eHealth literacy skills are generally sub par. College students, especially in the health and medical professions, would be well served to receive more customized college-level instruction that improves general eHealth literacy.

## Introduction

Electronic resources increasingly play a major role in consumer health, with the Internet being the preferred primary telecommunications vehicle for seekers of novel and germane health information. Although now widely relevant, the term electronic health information, also called *eHealth*, first appeared in 2000 to describe where health informatics, public health, health services, and information transmission processes intersected, primarily through Web-based applications [1,2]. Health information is one of the most investigated topics online [3]: 8 out of 10 Internet users report that they have at least once looked online for health information, making it the third most popular Web activity next to checking email and using search engines in terms of activities that almost everybody has done [4]. The importance of the Internet to acquire health information has spurred the creation of numerous eHealth information resources that assist consumers in discovering knowledge that can help promote and sustain personal health. Subsequent studies examining the effectiveness of eHealth interventions have proposed many definitions for eHealth [1,2,5]. Broadly stated, eHealth can also be thought of as the field where information and communication technology design enables the delivery of health-related and medical information [6]. While eHealth can potentially revolutionize medical and public health practice [7], numerous human resource, organizational, and cultural changes are still necessary to enable mainstream adoption of eHealth strategies for retrieving good-quality health information [1,8,9].

eHealth and the topic of *health literacy* are closely connected in public health. *Health literacy* is defined as “the degree to which individuals have the capacity to obtain, process, and understand basic health information and services needed to make appropriate health decisions” [10]. It has been identified as a public health goal for the 21st century and stands as a significant challenge facing health care globally [11,12]. According to Norman and Skinner, the articulation of health literacy “underscores the importance of contextual factors that mediate health information and the need to consider health literacy in relation to the medium by which health resources are presented” [13]. The pervasiveness of the Internet has made obtaining, processing, and understanding health information using Web-based technologies a critical competency area for medical professionals. With the emergence of electronic medical and health records, medical mobile apps, and other related health informatics technologies, medical professionals are increasingly responsible for finding and evaluating health information resources electronically. In light of this, *e* *Health literacy* now exists as an important skill set for health professionals tasked with seeking valid and reliable health information in a Web-based environment. However, most studies on literacy and health, such as the US Institute of Medicine’s report titled *Health Literacy: A Prescription to End Confusion*, exclusively examine the relationship between health outcomes and literacy in the context of paper-based resources, not literacy in electronic environments [11,13]. Therefore, *eHealth literacy* is still a novel concept with varied definitions and models.

*eHealth literacy* refers to the ability of individuals to seek, find, understand, and appraise health information from *electronic resources* and apply such knowledge to addressing or solving a health problem [13]. eHealth literacy combines six core skills or types of literacy: traditional literacy, health literacy, information literacy, scientific literacy, media literacy, and computer literacy [13,14]. Table 1 [12,13,15,16] provides definitions of each type of literacy considered within the scope of eHealth literacy. These six facets have been developed by Norman and Skinner and have been depicted as the *eHealth Literacy Lily Model*, characterizing the six types of literacy as forming overlapping lily petals that feed into the overall eHealth literacy “pistil” (ie, center of the model). More specifically, the lily model categorizes the six core literacies into two primary types: analytic (ie, traditional, media, and information) and context- specific (ie, health, scientific, and computer). Analytic literacies refer to a set of skills that can be applied to an array of information sources, whereas context-specific literacies involve skills that are specific to a certain problem or situation. eHealth literacy, as the composite of both analytic and context-specific skills, requires the behavioral capability to do the following: work with technology, critically think about issues of media and science, and navigate through the vast array of eHealth decision-making resources. A variety of competencies are associated with obtaining eHealth information, including the knowledge, skills, abilities, and other attributes necessary to (1) conduct basic and advanced information searches, (2) apply Boolean operators to limit searches, (3) differentiate between scholarly documents, authoritative sources, periodicals, and primary sources of information, and (4) understand sometimes ambiguous eHealth terminology. Specific techniques using these proficiencies are necessary to find documents on the Web such as abstracts, bibliographies, research articles, and government reports. To ensure that individuals are optimally making use of available eHealth access, it is important that appropriate search-related practices and procedures be used to retrieve and assess the eHealth information that is located.

**Table 1:** Six components of eHealth literacy[13]

Type of literacy	Definition
Traditional literacy	Involves basic literacy skills, such as reading text, understanding written passages, and coherently speaking and writing a language [15].
Information literacy	According to the American Library Association, involves a person knowing “how knowledge is organized, how to find information, and how to use information in such a way that others can learn from them” [16].
Media literacy	Involves the ability to critically think about media content, and “enables people to place information in a social and political context and to consider issues such as the marketplace, audience relations, and how media forms in themselves shape the message that gets conveyed” [13].
Health literacy	Defined by the American Medical Association as a person’s capability to “perform basic reading and numerical tasks required to function in the health care environment. Patients with adequate health literacy can read, understand, and act on health care information” [12].
Computer literacy	Involves the ability to use computers to solve problems. According to Norman and Skinner, “computer literacy includes the ability to adapt to new technologies and software and includes both absolute and relative access to eHealth resources” [13].
Scientific literacy	Involves an “understanding of the nature, aims, methods, applications, limitations, and politics of creating knowledge in a systematic manner” [13]. Allows health research findings to be placed in the appropriate context and requires the understanding of the discovery process.

Access to eHealth information is ubiquitous now for many who have broadband Internet; however, access to eHealth resources does not inevitably assure acuity in discerning good-quality health information from quackery on the Internet. The ability to diagnose and engage useful eHealth information from reputable medical sources, such as governmental agencies (eg, National Institutes of Health, Centers for Disease Control and Prevention, Health Canada) and medical establishments (eg, Mayo Clinic, WebMD, Canadian Medical Association) as compared with opinion or advertisements from so-called experts such as private sector marketers and nonverified public commentators, is becoming increasingly important. With the wealth of health information that exists on the Internet, this complex task requires far more interpretive and demonstrative skill than simply being able to enter a medical condition or term into an Internet search engine such as Google or Bing. For example, when using the Internet as a medical education resource, consumers should know how to critically examine and discriminate between primary and secondary sources of health information posted on a website [13]. 

Implementing effective Internet searches to locate health information is especially important for college students, as the Internet is now a favorite resource for information gathering among the “Millennial” generation. For the Millennial generation of college students, the Internet is a preferred source of health information [17]. While it may be safe to assume that college students have ample access to Web-based portals leading to eHealth information, it is important to be cognizant that access alone does not ensure that college students are adroit at searching for, locating, and evaluating health information. Ensuring that college students have the knowledge and skills necessary to conduct advanced eHealth searches is an important responsibility particularly for the medical education community. 

To determine eHealth literacy among college students, it is first important to define the specific knowledge, skills, abilities, and other user attributes that have been considered in previous eHealth literacy research. Some of these attributes have been investigated by Ivanitskaya and Casey, who used the Information Literacy Competency Standards for Higher Education, developed by the Association of College and Research Libraries [18,19], to create the Research Readiness Self-Assessment (RRSA). The RRSA measures basic information literacy skills related to research ability. Specific to measuring information literacy, the competency model assesses knowledge and skill sets necessary to locate good-quality information on a specific health topic. These competencies verify abilities to determine possible sources of good-quality health information, conduct health information searches, evaluate the quality of the information, and appropriately use the information. Declarative knowledge, such as knowledge of plagiarism, health-related information sources, and research terminology, consists of typical knowledge variables measured in this competency-based approach. In addition, procedural knowledge, which involves skills and problem solving, includes knowledge of the procedures used to complete an information-seeking task electronically (ie, database navigation). Both types of knowledge are important for assessing the behavior and eHealth literacy of health information consumers [19]. 

In summary, there is a growing interest in eHealth literacy as an essential skill for students, especially those in the medical and health professions, and it is unclear whether the current level of eHealth literacy is sufficient, or whether customized eHealth literacy training for finding, interpreting, and evaluating health- and medical-related information available on the Internet at the college level would be required to nurture these skills. The purpose of this systematic review is to evaluate the current literature to determine whether college students can generally be considered an “eHealth literate” population.

## Methods

### Search Procedures

This review adopted the widely accepted definition of *e* *Health literacy* as the ability of individuals to seek, find, understand, and appraise health information from electronic sources and apply such information to addressing or solving a health problem [13]. For this review, the experimental units of analysis for inclusion were peer-reviewed articles evaluating eHealth literacy (ie, seeking, finding, understanding, and appraising health information among electronic sources, primarily the Internet) exclusively among college students. The scope of the review was male and female college students between the ages of 17 and 26 years attending various 4-year colleges and universities located around the world. To generate a sample of empirical studies, we conducted an exhaustive search of electronic databases. Due to the relatively recent emergence of eHealth in the 21st century, only articles published from 2000 to the present day were eligible for inclusion. The actual search of all relevant literature took place during the spring of 2011. The searched databases were ERIC, PsycINFO, HealthSource, Medline, MasterFILE Premier, Academic Search Complete, CINAHL Plus with Full Text, Health Source: Nursing/Academic Edition, Psychology & Behavioral Sciences Collection, Applied Social Sciences Index and Abstracts, and CSA. The following key terms were entered in various combinations with multiple Boolean operators: *e* *Health*, *electronic health*, *eHealth literacy*, *electronic health literacy*, *health literacy*, *Internet literacy*, *Internet health*, *electronic literacy*, *college students*, *university students*, and *literature review*.

**Figure 1:**
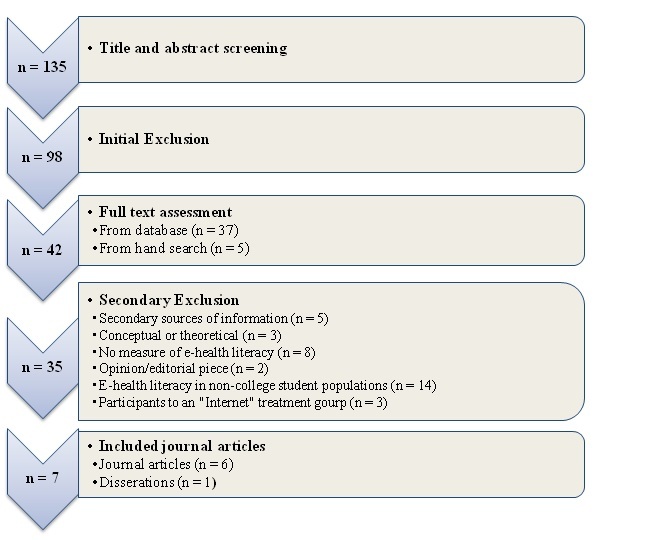
Flow diagram of the article selection process.

All articles gathered through this initial search and screen process (n = 135) were evaluated for inclusion in the sample pool. We excluded 98 records after the screen of titles and abstracts. We initially excluded studies that did not survey 4-year college student populations between the ages of 17 and 26 years, and eliminated those that did not measure knowledge, skills, abilities or other attributes associated with eHealth literacy. In addition to the 37 papers that remained after the initial exclusion, we identified 5 other articles by hand searches after scanning the reference section of each database-identified article to enhance the breadth of the examination. This hand search resulted in the addition of 5 other articles meeting criteria for a full-text assessment. Overall, 42 papers were included in this full-text assessment, of which 35 were excluded for a variety of reasons, including (1) being secondary sources of information (n = 5), or purely conceptual or theoretical in scope (n = 3), (2) acting as opinion or editorial pieces (n = 2), (3) including populations other than college students (n = 14), (4) not explicitly measuring and reporting students’ ability to seek, find, or evaluate electronic sources of health information (n = 8), or (5) reporting studies that assigned participants to an “Internet” treatment group within an intervention or trial (n = 3). After accounting for conditions outlined by the above exclusion criteria, we were left with 28 articles out of the review, leaving 7 articles that were empirical studies assessing eHealth literacy among college students. Figure 1 presents a flow diagram of the systematic literature review search process described above. Of the final 7 articles, 6 studies were carried out in the United States and 1 in Finland.

### Methodological Data Analysis

To evaluate the methodological quality of each retained study, we used a modified version of criteria established by Nagel Bernstein and Freeman [20] to develop a methodological data score (MDS) for each article ranging from 0 (low) to 4 (high). If a study used multivariate procedures such as discriminant analysis, factor analysis, cluster analysis, hierarchical regression, or multivariate analysis of variance, then it received a score of 4. Articles reporting descriptive statistics, univariate regressions, or nonparametric tests such as chi-square were assigned a 3. Those reporting strictly qualitative data received a score of 2, and purely narrative descriptions or written observations received a score of 1. When studies did not report any statistical analysis procedures, then no points were awarded.

## Results

### Study Characteristics

Although eHealth has been a topic of interest since the turn of the century, this systematic search identified only 6 peer-reviewed articles [19,21-25] and 1 doctoral dissertation [25], published between 2005 and 2010. Four different journals published the articles: *Journal of Medical Internet Research* (2 articles), *Journal of American College Health* (2), *BMC Medical Informatics and Decision Making*, and the *Californian Journal of Health Promotion*. A total of 4 articles [19,21-23] explicitly defined eHealth literacy, but all explored at least one aspect of eHealth literacy accounted for in the Norman and Skinner [13] definition used within this review. For example, Nsuangani and Perez [21] asked specific questions about Internet use tendencies to find health information, while the RRSA, administered in 3 studies [19,25,26], sought to evaluate all dimensions of eHealth literacy.

The studies included in this systematic review were generally exploratory in nature. Many used demographic variables to group students in a nonexperimental fashion and then explored differences in relationships. The independent variables used in all studies were unique and directly related to the study purpose; however, the most common independent variables were users and nonusers of the Internet, gender, student classification, and race. The dependent variables included self-reported use of Web-based health advice services [22]; perceptions of the accuracy of health information on the Internet [21]; perceptions of the privacy of health information on the Internet [21]; frequency of Internet use for seeking health information [20,22,23]; quality of health-related websites [19,25,26]; attitudes and beliefs about using the Internet for finding health information (eg, beliefs that open-access Internet and search engines are always the best sources of information) [25]; ability to find electronic health information [19,25,26]; ability to evaluate electronic health information [19,25,26]; perceived research skills (ie, self-reported subjective beliefs about one’s own skills) [19,25,26]; ability to judge the trustworthiness of Internet pharmacies [25]; and number of correct answers to sexual health questions following the conduct of Internet searches [24].

Results from the methodological assessment described above indicated that the reviewed articles had similar degrees of analytic rigor. That is, 2 studies randomly assigned participants to treatment groups [21,22], while the others used convenience [19,23,25,26] and purposive [24] sampling techniques; 6 of the studies [19,21-23,25,26] used a quantitative paradigm to determine patterns between independent and dependent variables, and 1 study [24] used a mixed-methods approach. Only 2 studies [21,26] used validated surveys containing reliability estimates for the data collected, while 3 studies [22,25,26] did not explicitly report this information. A total of 3 studies [21-23] used chi-square as the analysis of choice to explore differences in patterns between groups, and the remaining studies [19,24-26] simply reported descriptive statistics. Redmond [26] used multiple *t* tests to determine differences in eHealth literacy skills between rural and nonrural college students. Ivanitskaya and Brookins-Fisher [25] performed multiple independent *t* tests to assess whether differences in critical judgment existed among students who either did or did not use the Internet for health decision making. The mean MDS for the reviewed studies was 3.14 (SD 0.38), with 6 of the 7 studies (86%) scoring 3. Table 2 describes the basic design, measurement, and analysis of each study accompanied by each study’s individual MDS.

**Table 2:** Design, measurement, analysis, and methodological data score (MDS) of selected studies

First author (year)	Sample size	Design	Instrument	Instrument validity	Instrument reliability	Analysis	MDS
Ivanitskaya [19] (2006)	308	Nonexperimental	RRSA^a^	Face, content	Yes, but no value reported	Descriptive statistics, multiple regression	3
Nsuangani [21] (2006)	136	Experimental	Ad hoc survey	Face (expert panel)	Pre–post, κ = 0.41 for items retained for analysis	Frequency distributions; cross-tabulations; chi-square	3
Castren [22] (2008)	5030	Experimental	Student Health Survey 2004 (Finland)	Not reported	Not reported	Frequency distributions; cross-tabulations; chi-square	3
Escoffery [23] (2005)	743	Nonexperimental	Ad hoc survey	Not reported	Not reported	Descriptive statistics; chi-square	3
Buhi [24] (2009)	34	Nonexperimental	Ad hoc	Content (implied)	Not reported	Descriptive statistics	3
Ivanitskaya [25] (2010)	1914	Nonexperimental	RRSA	Face, content	Not reported	Descriptive statistics; probabilities; *t* tests; hierarchical regression analysis	4
Redmond [26] (2007)	243	Nonexperimental	RRSA	Face, content	Ability to obtain health information, alpha = .69; ability to evaluate electronic health information, alpha = .65; overall health information competency, alpha = .77	Descriptive statistics; *t* tests; Cohen d	3

^a^ Research Readiness Self-Assessment.

### Demographics

The number of participants in each reviewed study varied widely (ranging from 34 to 5030), which calls into question the representativeness of the results from those studies with smaller samples. Additionally, the research findings related to gender varied within 5 studies [19,21-23,26]. For example, Nsuangani and Perez [21] found that male college students were more likely to use the Internet to buy pharmaceutical products and locate consumer health information, whereas female students were more likely to obtain general health- and medical-related information online. Interestingly, this finding was supported in 2 other studies as well [22,23]. Males were more likely to seek out medical consultations using the Internet [21], while females were more likely to self-report diagnosing chronic health conditions using the Internet [22]. Based on this evidence, it appears that females used the Internet more for health information and diagnostic purposes, while males were more likely to use Internet for consumer health products and services. Also, male and female college students did not differ significantly as to whether they expressed concern regarding the accuracy of health information found on the Internet [21]. We found no statistically significant differences on any eHealth literacy outcome when considering race or ethnicity.

### Obtaining Health Information Using the Internet

We found that in 3 studies performed in the United States exploring the percentage of college students using the Internet to acquire health information, 91 of 136 (67%), 549 of 743 (73.9%), and 24 of 34 (71%) college students surveyed had ever used the Internet to search for health information. In 1 study, 111 of 743 (14.9%) college students reported using the Internet to locate health information in the past day or week, with less than one-third reporting doing so in the past month [23]. In the same study, 539 of 743 (72.5%) students reported being averse to logging onto a health program delivered over the Internet, and only 204 of 743 (27.5%) students surveyed reported willingness to participate in a health program on the Internet. Another study reported that participants were reluctant to use the Internet for interactive health purposes, with 119 of 136 surveyed students (87.5%) reporting an unwillingness to use online medical discussion applications [21]. Another study conducted in Finland corroborated this reticence to participate in online health programming, finding that only 370 of 3153 (11.7%) Finnish undergraduate students had ever used a Web-based health advice service offered to them through their student health services department [22]. 

While 1 study [23] suggested that 393 of 743 (52.9%) college students surveyed would like to individually retrieve health information on the Internet, several studies indicated college students self-reported a lack of skills necessary to execute successful health-related searches on the Internet [19,23,25,26]. Escoffery et al [23] noted that 661 of 743 (89.0%) college students surveyed did not always find their desired eHealth information. Among those, 82 (11%) students surveyed did feel that they were capable of finding health information on the Internet, whereas only slightly more than half reported success “most of the time.” Two studies [19,26] determined that many college students are rather unsophisticated health information seekers when using the Internet. Another study noted that college students were unable to critically evaluate health information found on the Internet [25]. Students were also unaware of the difference between a primary and secondary source of data when attempting to locate online journal articles in the health-related fields [19,26]. Finally, students who used eHealth information to help make health decisions had lower overall critical judgment ability than those who used nonelectronic sources of information for the same purpose [25]. 

### Perceived versus Actual eHealth Literacy

Ivanitskaya et al [19] and Redmond [26] assessed (1) how students felt about their own level of eHealth literacy, (2) how proficient students were at searching for and evaluating eHealth information, and (3) how well students understood the difference between peer-reviewed scholarly resources and opinion pieces or sales pitches. Both studies used the RRSA online assessment tool, which evaluated perceived and actual knowledge of student ability in browsing the Internet and researching health topics given various search scenarios. The RRSA, based on the Information Literacy Competency Standards for Higher Education, assesses knowledge and skills related to locating, evaluating, and using good-quality sources of eHealth information. Specifically, the RRSA contains the following items: “(1) multiple choice or true/false questions that measure declarative knowledge; (2) interactive, problem-based exercises that measure procedural knowledge; (3) demographic questions; and (4) a question that asks for a self-report about the level of the respondent’s research skills” [19]. For example, a knowledge-based item in the survey asks respondents to indicate which Boolean operator (eg, “and,” “or,” or “not”) produces the most Internet search results (answer: or). An example of a skill-based survey item asks respondents to determine which Boolean operator is appropriate for a particular search situation, then requests that the respondent perform an Internet search using that particular Boolean operator, followed by reporting back the number of Web hits generated by the search [27]. There are two subscale measurements within the RRSA: Actual Ability to Obtain (AAO) eHealth information and Actual Ability to Evaluate (AAE) eHealth information. The AAO subscale comprises 11 multiple choice items where total scores can range from 0 to 16. The AAE subscale comprises 13 multiple choice items where total scores can range from 0 to 23. A higher score on both subscales indicates better actual ability. One study within this review [19] demonstrated that the data derived from the RRSA possessed satisfactory internal reliability (alpha = .78). Ivanitskaya et al [19] found that 258 of 306 (84%) college students surveyed perceived their eHealth literacy skills as “good,” “very good,” or “excellent,” yet students’ scores on a 56-item scale evaluating their actual eHealth literacy skills were very poor (mean 37%, SD 6.4%).

Also, it was found that within each perceived skill category (eg, perceived ability to find health information and perceived ability to judge the quality of health information), the actual overall competency scores of college students varied greatly. Specifically, the ability of college students to evaluate their own competency was inconsistent with their actual eHealth literacy. Redmond [26] found that nonrural college students were better able to obtain eHealth information than were rural college students, but there were no statistically significant differences in the ability to evaluate eHealth information between the two groups. Escoffery et al [23] found that 260 of 743 (35%) college students surveyed expressed “serious concern” about their ability to find good-quality health information using the Internet, while only a small proportion, 52 of 743 (7%), expressed “no concern” regarding the accuracy of health information they acquired on the Internet. Despite the relatively higher level of apprehension regarding ability to find eHealth information, 204 of 514 (39.7%) college students who reported seeking health information online believed that being able to retrieve health information online improved the way they took care of their health “some” or “a lot.”

In light of these findings, all studies tended to agree that college students in general [19,21,23-26], and those in health and medical professional programs specifically [19,26], should further develop their proficiency in appraising, using, and evaluating health information found on the Internet. Table 3 describes the primary findings gathered from the research questions posed in each study.

**Table 3:** Primary findings from research questions

First author	Research question(s)	Findings
Ivanitskaya [19]	How proficient are university students at searching for health-related information?	Students are not proficient at advanced health information searches.
	How proficient are university students at evaluating health-related information?	Students have mixed proficiency at evaluating health-related information.
	How well do university students understand the difference between peer-reviewed scholarly resources, opinion pieces, and sales pitches?	Students are deficient in discriminating between different types information sources.
	How aware are university students of their own level of health information competencies?	Undergraduate students are inaccurate judges of their own health information competencies. Self-reports may not accurately predict students’ actual health information competencies.
Nsuangani [21]	Do male and female college students differ in their Internet behaviors related to health?	Males more likely than females to report online medical consultation. Males are more likely to buy pharmaceuticals online. More males use email to communicate with a health care provider.
Castren [22]	Does self-reporting of chronic conditions differ between users and nonusers of a Web-based health advice service?	Male users of a health advice service had a higher rate of self-reported chronic conditions than male nonusers; female users of a health advice service had a higher rate of a reported chronic condition than female nonusers.
Escoffery [23]	Are there differences in Internet use for health information by level of Internet experience?	There is no difference.
	Are there differences in Internet use for health information by gender?	Significantly more female than male students obtain health information online.
	Are there differences in Internet use for health information by level in college?	There is no difference.
Buhi [24]	When asked questions about sexual health, do college students find accurate answers online?	For 12 of the 13 questions asked, at least 24 of 34 (71%) students answered the questions correctly. Of 34 students surveyed, 17 (50%) correctly answered the question that asked them to locate an anonymous HIV test in the local area.
Ivanitskaya [25]	To what degree are college-educated information seekers able to determine trustworthiness of online pharmacies?	How college students rate trustworthiness of online pharmacies varies substantially. Only 593 of 1914 (31.0%) respondents gave low ratings to untrustworthy online pharmacies.
	Do those who used information to make health decisions have better judgment skills?	Respondents using online health information for decision-making have significantly worse judgment than those not using online health information for decision-making.
Redmond [26]	Do rural and nonrural freshmen differ in their ability to obtain health information?	A statistically significant difference exists, with nonrural students performing higher than rural students, *t*_241_ = 2.23, *P* = .03, Cohen d = .29.
	Do rural and nonrural freshmen differ in overall health information competency?	No difference exists, *t*_241_ = –.14, *P* = .89, Cohen d = .02.
	Do rural and nonrural freshmen differ in their ability to evaluate health information?	No difference exists, *t*_241_ = 1.34, *P* = .18, Cohen d = .18.

## Discussion

### Main Findings

The main conclusion of this systematic review was that college students may lack important skills for seeking and evaluating health information available on the Internet. While college students, for the most part, have convenient access to health information on the Internet, this systematic review indicated that many students possess weak eHealth literacy skills related to searching for, retrieving, using, and evaluating sources of eHealth information. Furthermore, 3 studies [19,25,26] noted that the subjective self-perceptions of college students regarding their ability to use eHealth information sources were incongruent with their demonstrated eHealth literacy skills. Therefore, it is possible that college students may be mistakenly judging their own ability to successfully locate and evaluate eHealth information. They may (or may not) hold an overly optimistic view of their ability to do Internet research on health-related topics. While it is clearly too early in this field of investigation to state definitively that there is a gap between perceived and actual eHealth literacy among college students, the trend noted in this systematic review provides impetus for future research to either support or disconfirm whether this phenomenon may truly exist.

Regardless of whether a discord exists between perceived and actual eHealth literacy among college students, there nevertheless is an invaluable opportunity to build medical education competencies among college-age students, especially those seeking degrees in the medical and health professions. College students surveyed in the reviewed studies did not achieve satisfactory levels of eHealth literacy; thus, we should perhaps reexamine the standards that are being used to measure eHealth literacy among this diverse audience. While valid and reliable *health* literacy measures have been widely established, far fewer instruments are universally accepted as accurately and appropriately assessing *eHealth* literacy. It is possible that the current standard being promulgated might be appropriate only for technologically elite audiences. Supposing that consumers will meet eHealth literacy standards set by technicians is probably unrealistic. High-stakes measures used in the current studies may have attempted to assess skill navigating the Internet to locate health information, but these measures may do so in a manner less applicable to a wide-ranging audience of future public health professionals. Future professionals, especially in a health-related field. will undoubtedly be using the Internet and related health informatics technologies to gather, manage, and deliver health information; however, we have yet to fully understand the context of the interactions occurring between diverse users and health informatics technologies (given that the consumer health informatics field is still in its infancy). There is a strong possibility that the broad-based, multidimensional definition of eHealth literacy is overly ambitious, even for individuals seeking an advanced degree in a health-related field.

While measurement issues are important to consider, it is also important to recognize that future inquiries should avoid reporting purely descriptive self-report data on frequency of use and self-efficacy for using the Internet to find health information. Data from self-reports depicting college students as both frequent and confident eHealth users may be more assumptive than truly substantive, especially considering the current research. As explained by Bandura within self-efficacy theory, “expectation alone will not produce desired performance if the [individual’s] component capabilities are lacking” [28]. Thus, what might be more important is testing of relevant skills in this new area of inquiry. Thus, more research should evaluate the most effective instructional strategies for molding able-bodied “eHealth educators” within a variety of medical and allied health professional preparation programs. Planned instructional experiences must consider the unique eHealth literacy competencies that are expected of college students studying to become health professionals, a distinction that places them in a unique position as compared with the general public. These students are expected to gravitate toward evidence-based practice and to critically appraise qualified sources of health information using specific resources such as The Cochrane Collaboration [29] or the Guide to Community Preventive Services [30]. More studies of college students at varied institutions, majoring in a variety of health and medical programs, will enable the eHealth literacy research community to ask and answer more targeted research questions with more specific audiences.

### Demographics

The literature also indicated a tendency for male college students to be more likely to use the Internet to locate and acquire consumer health products (eg, pharmaceuticals, dietary/sports supplements, vitamins, performance-enhancing substances) and services (eg, Web-based medical consultations) and less likely to search for general information on illness, disease, or disease prevention using medical reference websites. Female college students, on the other hand, were generally more likely to conduct these types of general health or medical searches and were less likely to obtain health services over the Internet (eg, accessing primary care physicians’ Web portals, communicating by email with health care providers). In light of this interesting preliminary trend, future research would benefit from further study regarding what particular Internet search and retrieval characteristics can be attributed to male or female college students. Unique search propensities could speak to various developmental issues of marketing pressures, peer influences, and even health privacy concerns.

### Obtaining Health Information Using the Internet

While the literature supports college students wanting to use the Internet to seek out general health information, there is little evidence to suggest that students care to discuss their own health problems or obtain personalized medical advice over the Internet. College students reported reluctance to using interactive Internet applications for health communication purposes (ie, electronic communication with health care providers). This finding revealed itself not only in the United States, but also in 1 Finnish study that we reviewed. Among college students, the convenience of using the Internet for *seeking* personal health information may be valued more so than the prospect of *receiving* individualized feedback on personal health concerns or problems via interaction with a qualified medical professional. This could be the result of contextual Web security issues affecting confidentiality. The issue of trust when using the Internet to seek and share medical information is an important one to consider, especially with the emergence of peer-to-peer or horizontal health communication among college students. More research should be done to discover what particular sources of Web-based health information college students are consulting and which cause uneasy feelings originating from potential threats to data security and privacy.

### Limitations

This systematic review had several limitations. Although we conducted a comprehensive literature search on numerous databases using a variety of pertinent search terms, certain studies may have been overlooked due to lack of indexing in searched databases. In addition, all studies were carried out in either the United States or Finland, which are both highly technologically savvy countries. Also, Finland is regarded as one of the world’s most literate societies, with high levels of educational attainment [31]. Another noted limitation is that one standard definition of eHealth does not exist, which limits the ability of researchers to find all articles examining eHealth literacy within a single literature review. Another limitation involves the number of articles included in the review. Although the studies reached similar conclusions in selected instances, the small sample of studies (n = 7) may not truly reflect the population’s (ie, college students) true eHealth literacy levels. In addition, most studies used convenience sampling techniques, which can result in findings not being reflective of the true populations of interest. As well, most studies in this review (n = 4) collected self-report data and did not test actual eHealth literacy skills to complement students’ self-perceptions.

The marketplace penetration of information technology into college students’ lives and educational settings is shifting rapidly (eg, smartphones, social networking websites, iPads). The reviewed studies of eHealth literacy among college students did not distinguish these emerging applications among the many alternative electronic sources of information, which may not truly reflect current search tendencies of college students. These types of applications conducive to mobile health information searches have spawned the new field of mHealth, which may suggest broadening or revising the study of eHealth literacy among college students. Finally, while the mean MDSs for the studies in this systematic review were quite good, few reported sufficient validity and reliability measures for data collected with survey or testing instruments, and almost all data analyses were univariate versus multivariate.

### Comparison With Prior Works

Even where access to basic Internet infrastructure exists or is provided, optimal Internet use is often limited by other factors, such as human interface. To some extent, human interface encompasses issues commonly considered when assessing *usability*. Usability of an eHealth information source typically refers to the quality of a user experience when interacting with the resource, with an emphasis on behavior rather than opinion or recollection [32,33]. The construct measures learnability, memorability, efficiency, frequency, and severity of errors. All of these aspects are affected by human limitations, such as literacy, and by health website quality criteria, such as accuracy, completeness, readability, and design. Thus, the construct of usability is inextricably linked with eHealth literacy. There are varying levels of usability among eHealth resources, so it would be useful to determine whether the perceived usability of resources is related to eHealth literacy outcomes [34,35]. An analysis that assesses individual perceptions of eHealth usability in relation to overall behavioral capability to locate and evaluate eHealth information is vital for future eHealth literacy research [33,35]. Studying consumer health informatics (ie, analyzing consumer needs for acquiring and using information retrieved using technology) in conjunction with eHealth literacy [34,35] can further develop methods that pave the way for providing health care service in the information age.

Consequently, collegiate degree programs for those entering the medical and allied health fields are uniquely positioned to nurture and develop eHealth competencies among future health professionals. It is important for education administrators to determine (1) what list of eHealth topics should be covered, (2) what types of courses and materials can address the needed competencies, (3) how many hours of subject matter instruction might be necessary for eHealth literacy skill development, and (4) whether eHealth warrants a specific emphasis area or track within professional preparation programs. Creating mission and policy statements that give attention to these relevant aspects of eHealth literacy instruction will help improve student outcomes.

### Conclusions

Evidence from this systematic review suggests that future health professionals need professional preparatory experiences that help build their eHealth literacy proficiencies. Enhanced skills development will likely develop as a product of practical medical Internet research opportunities that encourage critical thinking among students. As suggested by Escoffery et al [23], and supported by this systematic review, more needs to be done to inform the training of students in the health and medical professions, to “search the Internet for health information and to evaluate health information on Web sites.” Because of this, two important research questions should continue to be investigated in medical education. First, do professionally prepared college students in the health professions have the skills to navigate electronic resources to retrieve evidence-based health information? Second, do college students studying to be health professionals have an inflated sense of self-efficacy regarding their actual ability to locate and evaluate good-quality health information on the Internet?

Given that governmental and advisory agencies have designated eHealth literacy as paramount to improving societal health in both Canada [36] and the United States [37], it is important that future eHealth educators be provided with planned learning experiences in this growing field. Several health and medical disciplines have recognized this need area and have incorporated formal professional responsibilities related to eHealth literacy into core competency development models. For example, future professionals in the field of health education are expected to find valid health information resources electronically and evaluate the usefulness of such information [38]. It is important that health and medical education programs develop these types of proficiencies among future health professionals. Both current and future college students, especially those in the medical and health professions, need customized eHealth literacy training for finding, interpreting, and evaluating health- and medical-related information available on the Internet.

## Abbreviations

AAE: Actual Ability to Evaluate
AAO: Actual Ability to Obtain
